# Cost-effectiveness analysis of herpes zoster vaccination for adults aged 50 years in Zhejiang province, China

**DOI:** 10.3389/fpubh.2025.1401930

**Published:** 2025-05-13

**Authors:** Fuxing Chen, Linglin Ding, Yu Hu

**Affiliations:** Institute of Immunization and Prevention, Zhejiang Provincial Center for Disease Control and Prevention, Hangzhou, China

**Keywords:** cost-effectiveness, herpes zoster, adjuvanted recombinant zoster vaccine, live-attenuated zoster vaccine, vaccination

## Abstract

**Objective:**

Live-attenuated zoster vaccine (LZV) and adjuvanted recombinant zoster vaccine (RZV) have been approved for use in China. This study aimed to evaluate and compare the cost-effectiveness of these two herpes zoster (HZ) vaccination strategies in a birth cohort of individuals aged 50 years.

**Methods:**

The cost-effectiveness of RZV and LZV was compared to no vaccination strategy using a lifetime Markov model from a societal perspective. Model parameters were obtained from up-to-date published literature and statistical data. The costs associated with vaccination and medical treatment, quality-adjusted life years (QALYs), the number of herpes zoster and related complication cases averted, and the incremental cost-effectiveness ratio (ICER) were calculated. Sensitivity analyses were performed to assess the robustness of the results.

**Results:**

Compared to the no-vaccination strategy, the RZV strategy was estimated to prevent 4,126 HZ cases and 772 postherpetic neuralgia (PHN) cases, while the LZV strategy was estimated to avert 2,355 HZ cases and 467 PHN cases. The ICER was estimated at 711.46 US $/QALY and 914.62 US $/QALY for the RZV and LZV strategies, respectively.

**Conclusion:**

HZ vaccination could provide significant health benefits at a reasonable cost. RZV was predicted to be more cost-effective than LZV.

## Introduction

Varicella zoster virus (VZV) can manifest as either primary varicella or herpes zoster (HZ). Varicella typically affects children, whereas HZ results from the reactivation of latent VZV, leading to a recurrent disease state (1). The vesicular eruption associated with HZ typically presents unilaterally along the distribution of a sensory nerve. Postherpetic neuralgia (PHN), a distressing complication of HZ, can persist even after the lesions have resolved, and currently, no adequate therapy is available. PHN may persist for a year or longer following a zoster episode. Involvement of the ocular nerve and other organs can also occur with HZ, often leading to complications (2).

Specific T-cell-mediated immunity following primary infection with VZV helps prevent the virus from reactivating. Risk factors that weaken this cellular immune response cannot suppress the replication of VZV, thereby increasing the risk of developing HZ. Aging and immunosuppression are the most common risk factors (3). An earlier review reported that the incidence of HZ in the general population was 3–5/1000 person-years, increasing with age—from 6–8/1000 person-years at 60 years to 10–12/1000 person-years in individuals aged ≥80 years (4). The disability-adjusted life year (DALY) was 59.99 per 100,000 individuals, with the highest rates observed among those aged ≥50 years. Another review reported that the incidence was 5–8 per 1,000 individuals in the same age group, increasing to 11 per 1,000 among those aged ≥75 years (2). In addition to aging, female sex, immune deficiency, underlying diseases, and a family history of HZ have been identified as risk factors. A retrospective study examining the disease burden of HZ estimated that approximately 2.8 million new cases occur annually in China (5).

Vaccination is widely regarded as the most effective intervention for reducing the incidence of HZ. Two licensed vaccines for HZ are available in mainland China, namely adjuvanted recombinant zoster vaccine (RZV) and live-attenuated zoster vaccine (LZV). RZV is recommended for individuals aged ≥50 years and is administered in a two-dose schedule, with an interval of 2–6 months between doses. In contrast, LZV is suitable for adults aged ≥40 years, with a one-dose schedule. The overall vaccine efficacy (VE) is 97.20% for RZV (6) and 57.62% for LZV, according to the package insert. However, the willingness to receive the HZ vaccine remains suboptimal, with only 16% of respondents expressing a willingness to receive RZV, based on a survey conducted in China (7).

Both HZ vaccines are voluntary vaccines and are not currently included in the national immunization program. The main reason for this exclusion is the lack of health economics evaluation data. Indeed, the cost-effectiveness analysis of these two HZ vaccines is limited within the Chinese context. Evaluating the health and financial implications of vaccine implementation is crucial for informed public health policy decisions (8). Furthermore, comparing RZV and LZV is also important as it can help in priority setting and strategic planning, especially when operating under a constrained budget. This study aimed to evaluate the cost-effectiveness of HZ vaccination in a birth cohort of individuals aged 50 years from a societal perspective, as well as to compare the two strategies: RZV and LZV.

## Methods

### Study design

A decision tree-Markov model was used to assess the cost-effectiveness of introducing RZV and LZV into the immunization program in Zhejiang province, with the no-vaccination strategy as the baseline. The model evaluated the clinical and economic outcomes over the full life cycle for a target cohort of 10,67,006 adults (9), adopting a societal perspective. Treeage Pro 2022 was used for model construction and data analysis.

**Figure 1:**
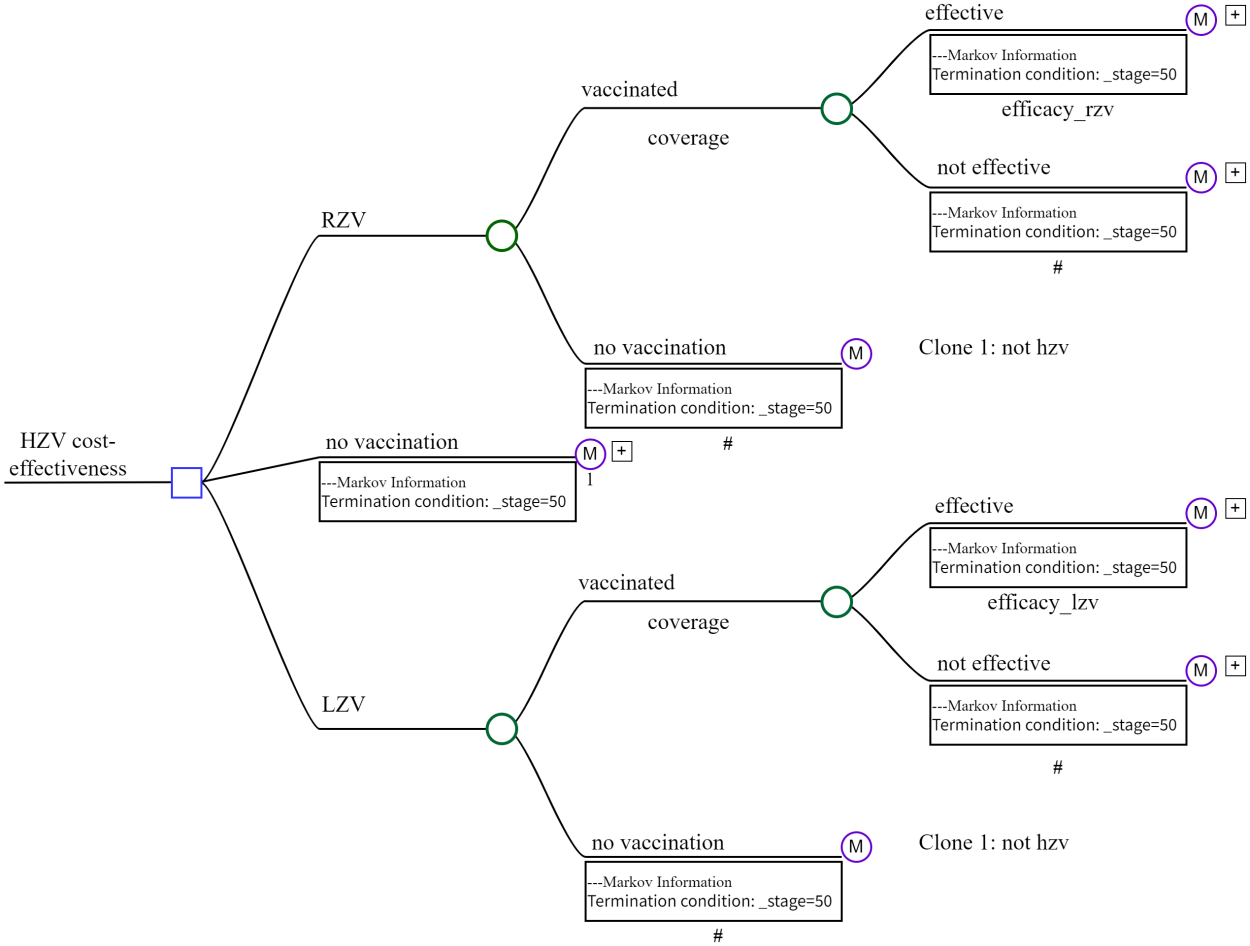
Overview of the decision tree-Markov model.

Based on the natural history of HZ, each individual in the cohort was initially categorized as being in a healthy state and then transitioned to other states over a one-year cycle within a 50-year lifetime horizon. In our model, HZ development was considered with or without complications, such as neurological diseases, PHN, ocular complications, or auricular complications. Individuals who recovered from HZ and its complications might experience a relapse into recurrent HZ if they transition through the state of resolved HZ (Figure 1). Both natural incidence and vaccine efficacy could influence the probability of each disease state.

We applied a half-cycle correction and set the Markov state cycle length to 1 year. The willingness-to-pay (WTP) threshold, equivalent to one times the gross domestic product (GDP) per capita of Zhejiang province in 2022 (16865.70US $) (9), was utilized for sensitivity analysis. No vaccination was defined as the control scenario. Given the relatively low risks of adverse reactions associated with the two HZ vaccines, these reactions were not considered in this analysis. Assuming that HZ vaccines would be included and funded by the local government, a coverage rate of 90% was also assumed, similar to other vaccines funded by the government (10).

### Model parameters

The parameters in the model were derived from the latest literature, statistical data, and the opinions of local experts. A team of 10 experts with diverse backgrounds in infectious diseases, epidemiology, immunization program management, vaccinology, health economics, and public health was assembled. When multiple data sources were available, the data that best fit the screening criteria, domestic data or local data, were selected. Sensitivity analyses were conducted using the low and high ranges of the parameters. In cases where the parameter range was unavailable, a board range was used by either subtracting 20% from the base value or adding 20% to the parameter’s value. Table 1 presents the parameters in the model, along with the sensitivity analysis range and distribution.

**Table 1:** Parameters used in this model.

Parameters	Age (year)	Base value	Range	Distribution	Source
Birth cohort	50	1,067,006	-		(9)
Incidence (/100,000)					
HZ incidence	50–59	0.08	±20%	B	(11)
	60–69	0.10	±20%		(11)
	≥70	0.15	±20%		(11)
Recurrent rate	50–59	0.12	-		(12)
	60–69	0.17	-		(12)
	≥70	0.28	-		(12)
Natural mortality	50–59	161.01	-		(9)
	60–69	398.19	-		(9)
	70–79	2097.85	-		(9)
	≥80	13555.25	-		(9)
Mortality of HZ	50–59	0.70	-		(13)
	60–69	1.30	-		(13)
	70–79	3.60	-		(13)
	≥80	31.30	-		(13)
Proportion (%)					
Hospitalization of uncomplicated HZ	50–59	1.40	1.12–1.68	T	(15)
	60–79	3.90	3.28–4.68		(15)
	≥80	8.10	6.48–9.72		(15)
PHN	50–59	7.00	5.66–8.48	T	(14)
	60–79	14.48	12.34–18.50		(14)
	≥80	26.72	19.52–37.84		(14)
Ocular	50–59	0.92	0.74–1.10	T	(15)
	60–79	0.69	0.55–0.83		(15)
	≥80	0.41	0.33–0.49		(15)
Auricular	50–59	1.48	1.18–1.98	T	(15)
	60–79	2.01	1.61–2.41		(15)
	≥80	1.42	1.14–1.70		(15)
Neurological	50–59	0.26	0.21–0.31	T	(15)
	60–79	0.29	0.23–0.35		(15)
	≥80	0.20	0.16–0.24		(15)
Cost (US $)					
Full-series of RZV		443.90	±20%	U	Local data
Full-series of LZV		190.14	±20%	U	Local data
Vaccination service(/dose)		3.89	±20%	U	Local data
Uncomplicated HZ (outpatient)		312.40	±20%	L	(17)
Uncomplicated HZ (inpatient)		1756.95	±20%	L	(17)
PHN		6820.29	±20%	L	(18)
Ocular		1042.40	±20%	L	(19)
Auricular		1308.16	±20%	L	(19)
Neurological		12821.54	±20%	L	(20)
Utility					
Lenth of HZ (month)		1	±20%	B	(21)
Lenth of PHN (month)	50–69	8.30	±20%	B	(21)
	≥70	10.90	±20%		(21)
Utility score of health		1	-		(22)
Utility score of uncomplicated HZ		0.79	0.68–0.88	B	(22)
Utility score of PHN		0.55	0.44–0.66	B	(22)
Utility score of ocular		0.67	0.54–0.74	B	(22)
Utility score of auricular		0.52	0.42–0.63	B	(23)
Utility score of neurological		0.20	0.10–0.50	B	(24)
Coverage for full-series		0.90	0.85–0.95	U	Local data
Efficacy for full-series					
RZV	50–59	0.97	0.94–0.99	B	Package insert
	60–69	0.97	0.90–0.99		Package insert
	70–79	0.91	0.86–0.95		Package insert
	≥80	0.91	0.80–0.97		Package insert
LZV	50–59	0.63	0.40–0.78	B	Package insert
	60–69	0.64	0.43–0.79		Package insert
	≥70	0.19	0.00–0.64		Package insert
Discount rate		0.03	0.01–0.05	U	(16)
Vaccine wastage rate		0.03	0.01–0.05	U	Local data

The population size of the target cohort and the age-specific all-cause mortality rate were derived from the 2022 Statistical Yearbook of Zhejiang province (9). The age-specific incidence of HZ was derived from a Chinese report (11). The incidence of recurrent HZ was obtained from a disease burden study on HZ (12). The mortality rate of HZ was sourced from a report in Japan, given the similarity in disease patterns between the two countries (13). The age-specific proportions of HZ cases with PHN were extracted from an epidemiologic study conducted in Taiwan (14). The age-specific proportions of HZ cases with other complications and the hospitalization rate for HZ were obtained from an epidemiologic study on HZ in Yichang, China (15).

Costs were reported in US dollars, with an exchange rate of 1 US $ = 7.20 RMB in 2023. These costs were discounted annually at a rate of 3% (16). According to the existing vaccination service system, the vaccination cost per dose included the vaccine cost (US $190.14 for LZV and US $221.95 for RZV) and management cost (US $3.89). Additional costs for manpower and fixtures were not considered. The vaccine wastage rate was set at 3%. The average cost of outpatient and inpatient visits for uncomplicated HZ cases was obtained from a disease burden report on HZ (17). The cost of PHN was derived from a Chinese study that gathered data from physician reviews and patient reports in eight hospitals (18). The expenses associated with ocular and auricular complications were based on the Chinese Health Statistical Yearbook 2021 (19). Due to the unavailability of specific cost parameters for neurological complications, we used financial burden data for meningitis in China as a proxy (20).

The health outcome of the model was measured in quality-adjusted life years (QALYs). The duration of HZ was assumed to be 1 month, as reported in an HZ prevention study for LZV (21). For individuals aged 50–69 years and those aged ≥70 years, the duration of PHN was set at 8.30 months and 10.90 months, respectively, according to the same study (21). The utility scores for PHN, uncomplicated HZ, and ocular complications were derived from a health-related quality of life study on PHN and HZ (22). The utility scores for auricular and neurological complications were obtained from a study on the disease burden of otitis media in older adults from Taiwan and a cost-effectiveness study of meningitis from China, respectively (23, 24).

The vaccine efficacy of the HZ vaccines was extracted from the package inserts of LZV and RZV. Herd effects of PCV13-TT against SP disease were not considered in this study due to the lack of conclusive data.

### Analytic framework

The predominant outcomes of the study included lifetime costs and QALYs obtained under different strategies. Compared to the no-vaccination strategy, the incremental cost-effectiveness ratio (ICER) for RZV and LZV was calculated using the following formula: (Cost_HZ vaccine_ − Cost_no-vaccination_)/(QALY_HZ vaccine_ − QALY_no-vaccination_) (25). The secondary outcomes included the health impact in terms of total cases of HZ, PHN, other complications, and HZ-related deaths averted under each strategy.

To assess the robustness of the model, a sensitivity analysis was performed. Specifically, a probabilistic sensitivity analysis (PSA) was performed by simultaneously varying model inputs according to their specified distributions across 10,000 Monte Carlo simulations.

## Results

Under the different HZ vaccination strategies, the cumulative reductions in HZ cases, complications, and deaths for the 2022 cohort of individuals aged 50 years are presented in Table 2. Compared to the no-vaccination strategy, full-series vaccination with RZV was estimated to avert 4,126 HZ cases, 772 PHN cases, 28 ocular complications, 58 auricular complications, 9 neurological complications, and 2 HZ-related deaths. In contrast, full-series vaccination with LZV was estimated to avert 2,355 HZ cases, 467 PHN cases, 15 ocular complications, 34 auricular complications, and 6 neurological complications.

**Table 2:** Base-case results of herpes zoster vaccination versus no vaccination.

Parameter	Scenario		
	No-vaccination	LZV	RZV
Averted disease events			
HZ case	-	2,355	4,126
PHN case	-	467	772
Auricular complications	-	34	58
Ocular complications	-	15	28
Neurologic complications	-	6	9
HZ related deaths	-	1	2
Cost (million US $)			
Costs	9526.44	14113.19	14779.62
Incremental cost	-	4586.75	5253.18
Effectiveness (QALY)			
QALYs(million)	8.14	13.16	15.52
Incremental effectiveness (million QALY)	-	5.01	7.38
Incremental C/E		914.62	711.46

Cohort size: 10,67,006; vaccination coverage for full-series: 90%; discount rate: 5%.

Table 2 also presents the costs of the different vaccination strategies: no vaccination, LZV, and RZV. Compared to the no-vaccination strategy, the cost of gaining one QALY among the individuals aged 50 years was 914.62 US $ for the LZV strategy and 711.46 US $ for the RZV strategy.

**Figure 2:**
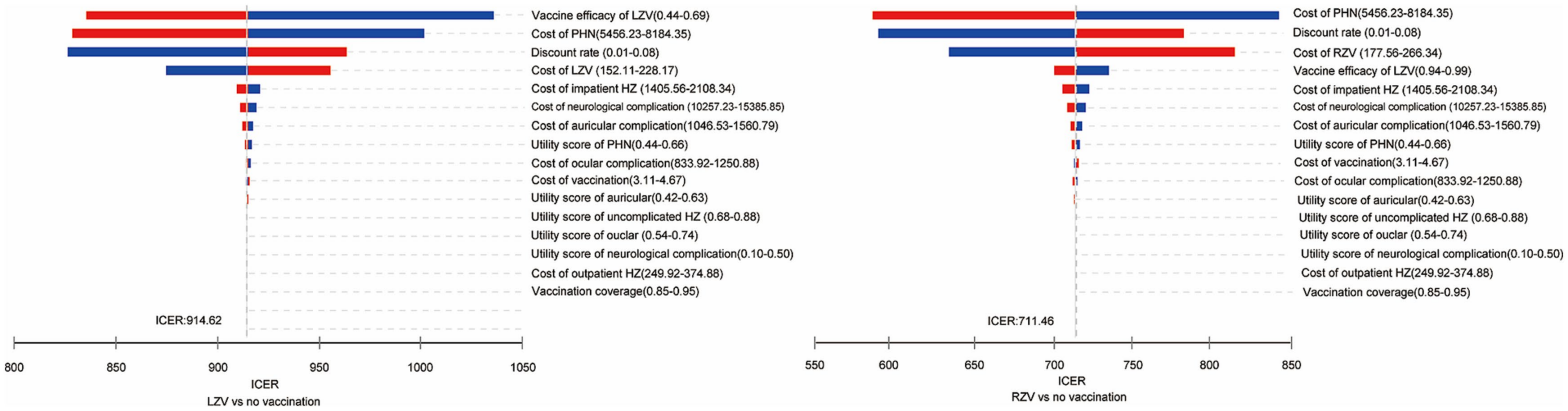
One-way sensitivity analysis of the cost-effectiveness of the herpes zoster vaccines compared to the no-vaccination strategy in adults aged 50 years.

One-way sensitivity analysis was performed to identify the most sensitive parameters, and the results are presented as tornado diagrams (Figure 2). Parameters such as vaccine efficacy, healthcare cost of PHN, and cost of vaccine significantly affected the results of the ICER for the LZV strategy. Parameters such as the healthcare cost of PHN, discount rate, and cost of vaccine significantly affected the results of the ICER for the RZV strategy.

**Figure 3:**
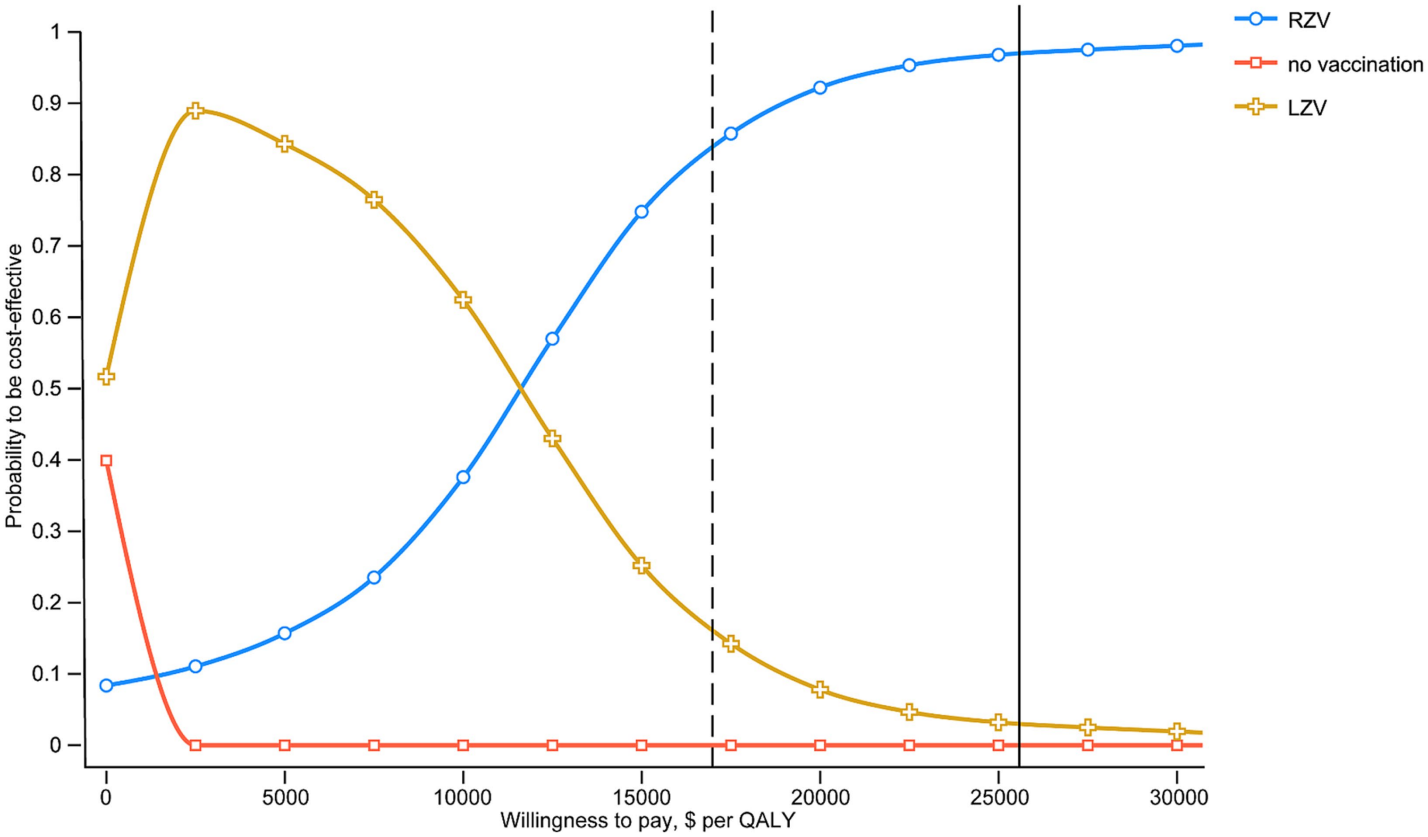
Probability of HZV and the no-vaccination strategy being cost-effective across a wide range of WTP thresholds. The solid and dashed vertical lines indicate 1-fold and 1.5-fold GDP per capita ($16865.70 and $ 25297.50), respectively.

Acceptability curves were constructed to examine the probability of being cost-effective for each vaccination strategy using a wide WTP threshold of $0–60,000/QALY (Figure 3). The probabilities of being cost-effective were 83.79% for RZV, 16.21% for LZV, and 0.00% for the no-vaccination strategy, at a threshold equivalent to one times the GDP per capita.

## Discussion

To the best of our knowledge, this study is the first health economic assessment of the health effects and cost-effectiveness of different types of HZ vaccines conducted in Zhejiang, China. Our findings provide reliable evidence that can assist public health decision-makers in improving the acceptance and vaccination coverage of HZ vaccines in Zhejiang province. Compared to no vaccination, both RZV and LZV could prevent thousands of HZ cases and hundreds of related complications. Although both types of HZ vaccines were cost-effective for adults aged 50 years and above, under a WTP threshold equivalent to one times the GDP per capita, RZV provides greater health benefits than LZV, with a lower ICER of 711.46 US $/QALY, making it the more cost-effective option. Our findings are consistent with those of previous health economic studies on HZ vaccination conducted in the Netherlands and the U.S. (26, 27), suggesting that vaccination with either HZ vaccine could be cost-effective. However, RZV appears to be more cost-effective, even when priced similarly.

In China, the current price for a full series of RZV is 443.90 US $, whereas in developed countries, the price ranges from 223 to 280 US $ (13, 26, 27). Since RZV was the first HZ vaccine approved in China, the pharmaceutical company set a higher price to offset the risk of the promotion cost associated with entering a new market and the uncertainty of demand. In 2023, LZV was approved for marketing in China at a lower price of 190.14 US $ for a full series. A lower cost of vaccine might be more feasible through government financial reimbursement or direct inclusion in the immunization program. However, the vaccine efficacy of LZV is not as optimal as that of RZV, which resulted in a higher ICER in our study. It is worth noting that potential market competition between the two HZ vaccines could lead to a further reduction in vaccine prices, potentially improving the acceptance of HZ vaccination among older adults.

The findings remained robust throughout the sensitivity analysis. The cost-effectiveness of the HZ vaccines was mostly influenced by vaccine efficacy, vaccine cost, the healthcare cost of PHN, and the discount rate. These factors align with previous findings (28, 29). In the one-way sensitivity analysis, vaccine efficacy was identified as an important parameter in most vaccine health economic evaluations. As vaccine efficacy decreased, the risk of HZ in vaccinated individuals increased, which, in turn, decreased the QALYs gained and increased healthcare costs. Initially, the high vaccine efficacy of RZV, which was at least 90% even in adults aged 70 years and older, made it more cost-effective, allowing it to command a substantial premium over LZV. Similarly, when the cost of vaccine or healthcare for PHN decreased, the incremental cost of vaccination also decreased. Consequently, both RZV and LZV might be more cost-effective. A reduction in the discount rate resulted in a marginal increase in QALYs while reducing the incremental costs associated with the vaccine and vaccination process.

Our study has several strengths. First, it provides a distinctive analysis of the efficacy and cost-effectiveness of both LZV and RZV within the context of Zhejiang province. Second, our findings align with previous economic evaluations of HZ vaccines conducted in various regions. Third, our modeling approach incorporated comprehensive predictions that account for the variability in HZ incidence and disease burden. Finally, extensive sensitivity analyses validated the robustness of our findings.

There are several limitations. First, we did not consider the long-term vaccine efficacy, as estimates of the waning rate of immunity varied across different studies. Second, exposure to VZV could provide protection against HZ in adults residing with children. Furthermore, the implementation of widespread varicella vaccination programs for children could potentially increase the incidence of HZ in adults due to a decrease in exogenous boosting effects (30). However, this study did not consider the potential influence of varicella vaccination on HZ incidence (31). Third, the impact of severe adverse events following the HZ vaccine was not considered in the model. Since the coverage of both LZV and RZV and the incidence of severe adverse events were low, the additional cost and QALYs might not be impacted by these factors. Fourth, only one type of neurological complication was included in this study, which might underestimate the true disease burden.

## Conclusion

Our analysis suggests that vaccination against HZ is likely a cost-effective intervention. However, vaccination with RZV is predicted to provide greater benefits and is likely to be more cost-effective than LZV. Based on the cost-effectiveness analysis and findings outlined in this study, we recommend that individuals aged 50 years and older receive RZV.

## Data Availability

All data generated or analyzed during this study are included in this published article. Requests to access the datasets should be directed to husix@163.com.
